# Domestic animal genetics

**DOI:** 10.1371/journal.pgen.1009831

**Published:** 2021-10-18

**Authors:** Tosso Leeb

**Affiliations:** Institute of Genetics, University of Bern, Bern, Switzerland; HudsonAlpha Institute for Biotechnology, UNITED STATES

## Introduction

Domestication of wild animal and plant species may be considered the largest genetic experiment in human history. Human selection for specific traits and targeted breeding over hundreds or thousands of generations has shaped very special populations that provide unique opportunities for research. Domestic animal genetics contributes to technological advances facilitating a more sustainable, environment and climate friendly food production that respects changing societal expectations related to ethical and animal welfare issues. Domestic animal genetics also enables fundamental insights into gene functions and provides animal models for biomedical research. Under this theme, we present a selection of *PLOS Genetics* publications that exemplify recent progress in domestic animal genetics. Rather than grouping by species, we assigned the publications to 5 thematically related topics, which we will briefly introduce in the following sections.

### Molecular tools, computational methods, and resources for domestic animal genetics

The 7 articles in this group provide either new methods or resources for research in domestic animal genetics. This ranges from an improved cat reference genome assembly over the generation of a comprehensive gene expression atlas in sheep to the presentation of a comprehensive database on breed-specific disease allele frequencies in dogs.

### Domestication genetics

The genetic changes associated with domestication show many parallels across different animal species, such as specific changes in behavior or the loss of seasonality of reproduction. In our collection, we present 8 articles that investigate specific genetic changes that are the consequence of breeding in closed populations or that investigate admixture and subsequent selection mechanisms in domestic animals.

### Complex traits

Nine articles in this section nicely reflect different levels of genetic complexity. They range from oligogenic traits to very complex traits such as the influence of the host genome on rumen microbiome diversity. In many livestock populations, a wealth of environmental and phenotypic data is recorded. This enables powerful studies with respect to many complex traits and genotype x environment interactions. With the intensive use of artificial insemination, elite livestock sires can have many thousands of offspring, which is a unique situation in mammalian species and predestines livestock populations for genetic research. When it comes to disease genetics, dogs with their many breed-specific predispositions for specific diseases provide excellent opportunities to identify genetic risk factors for complex diseases.

### Monogenic traits, new genotype–phenotype correlations, and *in vivo* function of genes

With 23 articles, this is by far the largest group in our collection. The special population structure of domestic animals greatly facilitates the identification of the causative genetic variants for hereditary traits. At the same time, sophisticated veterinary diagnostics approaching the standards in human medicine is widely available for cats, dogs, and also other domestic animals. Purebred animals are maintained in strictly closed populations with a substantial amount of inbreeding, which favors the emergence of new recessive traits and diseases. The elucidation of the causative genetic variants in spontaneous domestic animal mutants may assign physiological functions to uncharacterized genes, provides candidate genes for human medical genetics, and helps to obtain more comprehensive genotype–phenotype correlations.

### Cancer genetics

Dogs and also cats may develop very similar tumors as humans. The shorter life span and striking breed predispositions for certain cancer types facilitate the identification of genetic risk factors. With 5 articles, this is the smallest group in our collection. However, the potential of domestic animals and especially dogs for cancer research in general and cancer genetics in particular has by far not yet been fully utilized.

## Conclusions

*PLOS Genetics* continues to publish articles with some of the best research in domestic animal genetics. This is a field with many facets enabling fascinating new fundamental insights as well as laying the basis for applications in animal breeding that are absolutely vital for a sustainable future of mankind.

